# Complete Genome Sequence of Levilactobacillus brevis Bacteriophage ENFP1

**DOI:** 10.1128/mra.00203-22

**Published:** 2022-06-09

**Authors:** Jena Kim, Minsuk Kong

**Affiliations:** a Department of Food Science and Technology, Seoul National University of Science and Technology, Seoul, South Korea; Portland State University

## Abstract

We describe the complete genome sequence of bacteriophage ENFP1, which infects Levilactobacillus brevis; it has a capsid width of 83 nm and a tail length of 144 nm. The 138.6-kb genome, containing 190 predicted protein-coding genes, is similar (88.03% nucleotide sequence identity) to that of L. brevis phage 521B.

## ANNOUNCEMENT

Levilactobacillus brevis, a Gram-positive lactic acid bacterium, is one of the most common causes of beer spoilage (1, 2). Due to the increasing demand for natural food preservatives, bacteriophages possess the potential to control such spoilage bacteria (3, 4). Here, we sequenced the genome of the L. brevis bacteriophage ENFP1 isolated from a sludge sample collected at the Jungnang Water Reclamation Center in Seoul, South Korea.

Levilactobacillus brevis KACC 11433 was used as the host bacterium for the isolation and propagation of phage ENFP1. The bacteria and phage were grown in lactobacilli de Man, Rogosa, and Sharpe (MRS) broth (BD Difco) at 30°C. To isolate a bacteriophage, we used the same method as in the previous study (5) with some modifications. In brief, 100 μL of a host bacterial overnight culture was added to 5 mL of MRS soft agar (0.7% agar) containing 5 mM MgCl_2_ and CaCl_2_, and this mixture was poured onto MRS agar plates (1.5% agar). Then, 10 μL of a sample was spotted onto the bacterial lawn and incubated until plaques were visible. After a single plaque was purified three times, phage propagation and purification were performed as previously described (6).

To isolate the genomic DNA of phage ENFP1, phenol-chloroform extraction was used (6). The Illumina TruSeq Nano DNA library prep kit was used to create the sequencing libraries. The phage genome was then sequenced using the Illumina MiSeq platform. A total of 2,148,124 raw reads were obtained with read lengths of 300 bp (paired-end format). The short-read sequence data were assembled using Unicycler v.0.4.8. Every assembly was performed after quality filtering, and quality control steps were performed through two processes. At first, fastqc was carried out to measure base quality per cycle. After checking them, trimmomatic was conducted with these parameters (leading: 3, trailing: 3, slidingwindow 4:30, minlen 200) to remove low-quality bases and reads (7). The average genome coverage was 586.69×. The phage genome termini were identified with PhageTerm (8). BLASTP, InterProScan, and the NCBI Conserved Domain database (CDD) were used to analyze conserved protein domains (9–11). To predict tRNAs, the tRNAscan-SE 2.0 server was used (12). The presence of virulence factors in the genome was observed with the virulence factor database (VFDB) (13). All tools were run with default parameters unless otherwise specified. Bacteriophage ENFP1 was observed using energy-filtering transmission electron microscopy (EF-TEM) at the National Instrumentation Center for Environmental Management after being negatively stained with 2% (wt/vol) uranyl acetate (Seoul, South Korea).

**FIG 1:**
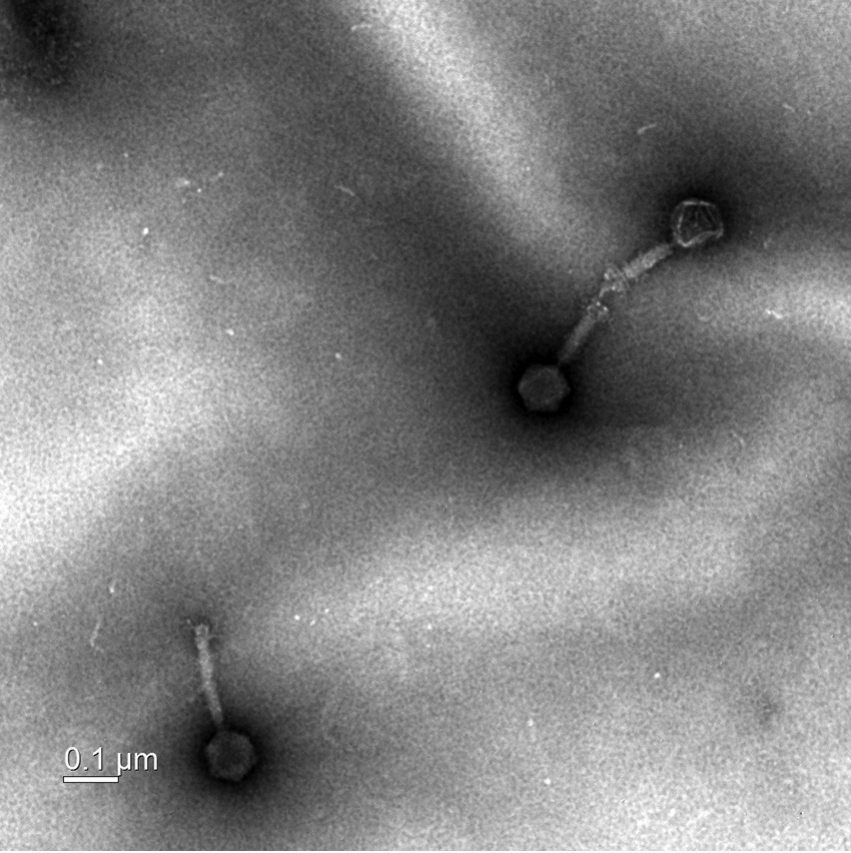
A transmission electron microscopy (TEM) image of L. brevis phage ENFP1.

Bacteriophage ENFP1 has a large icosahedral head of 83.25 ± 6.10 nm (mean ± standard deviation; *n* = 8) in diameter with a contractile tail of 143.9 ± 34.54 nm (*n* = 10) (Fig. 1). Based on its TEM morphology, ENFP1 appears to belong to the *Myoviridae* family in the order *Caudovirales* (14). ENFP1 contains a linear, double-stranded DNA genome of 138,676 bp with a G+C content of 32.5%, and it has T5-type long terminal repeats (6,973 bp) at both ends. Genome annotation revealed that it contains 190 predicted protein-coding genes, including several genes predicted to be involved in nucleotide metabolism (a DNA polymerase, a bacterial DNA-binding protein, a DNA primase, and a DNA helicase), structural proteins (a capsid protein, a major capsid protein, a baseplate protein, a tail sheath protein, and a tail fiber protein), host lysis (a holin and an endolysin), and 11 tRNA genes. There are no genes related to virulence factors. Considering that ENFP1 formed clear plaques and does not have lysogeny-related genes (e.g., integrases or repressors) in its genome, we assume that phage ENFP1 is a lytic phage.

BLASTN analysis with standard nucleotide collection databases showed that L. brevis phage 521B (GenBank accession no. NC048752) and L. brevis phage SAC12B (GenBank accession no. NC048754) isolated from L. brevis non-beer-spoiling strains are the closest relatives, with 88.03% genome sequence identity in 72% query cover and 90.80% identity in 73% query cover, respectively (1).

### Data availability.

The genome sequence of Levilactobacillus brevis phage ENFP1 has been submitted to the NCBI database under the accession number OM293948, BioProject accession number PRJNA799921, BioSample accession number SAMN25220521, and SRA accession number SRP357036.
